# Determinants of Nutritional Risk among Community-Dwelling Older Adults with Social Support

**DOI:** 10.3390/nu15112506

**Published:** 2023-05-28

**Authors:** Susana Ganhão-Arranhado, Rui Poínhos, Sílvia Pinhão

**Affiliations:** 1 CINTESIS, Centre for Health Technology and Services Research, 4200-450 Porto, Portugal; 2 Atlântica, Instituto Universitário, Fábrica da Pólvora de Barcarena, 2730-036 Barcarena, Portugal; 3 Faculty of Nutrition and Food Sciences, Universidade do Porto, 4150-180 Porto, Portugal; ruipoinhos@fcna.up.pt (R.P.); silviapinhao@fcna.up.pt (S.P.); 4 Serviço de Nutrição do Centro Hospitalar Universitário de São João, E.P.E, Alameda Prof. Hernâni Monteiro, 4200-319 Porto, Portugal

**Keywords:** older adults, nutritional status, malnutrition, risk, health conditions

## Abstract

Background: It is well established that older adults are at risk for malnutrition due to several social and non-social determinants, namely physiological, psychosocial, dietary and environmental determinants. The progression to malnutrition is often insidious and undetected. Thus, nutritional assessment should consider a complex web of factors that can impact nutritional status (NS). The primary objective of this study was to assess the NS of older adults attending senior centres (SCs) and to identify its predictors. Methods: This cross-sectional study enrolled a sample of community-dwelling older adults in Lisbon. NS was assessed using Mini Nutritional Assessment (MNA^®^). Malnutrition or malnutrition risk (recategorised into a single group) was predicted using binary logistic regression models, considering those participants classified as having a normal NS as the reference group. Data were collected through face-to-face interviews and anthropometric indices were measured according to Isak procedures. Results: A sample of 337 older adults, with an average age of 78.4 years old (range 66–99), mostly women (*n* = 210; 62.3%), were enrolled. Older adults at risk of malnutrition accounted for 40.7% of the sample. Being older (OR = 1.045, CI 95% [1.003–1.089], *p* = 0.037), having a worse perception of health status (OR = 3.395, CI 95% [1.182–9.746], *p* = 0.023), having or having had depression (OR = 5.138, CI 95% [2.869–9.201], *p* < 0.001), and not having or having had respiratory tract problems (OR = 0.477, CI 95% [0.246–0.925], *p* = 0.028) were independent predictors of malnutrition or malnutrition risk. An intermediate time of SC attendance was associated with a lower probability of malnutrition or risk (OR = 0.367, CI 95% [0.191–0.705], *p* = 0.003). Conclusions: NS among older adults has a multifactorial aetiology, with a strongly social component and is related to health circumstances. Further research is needed to timely identify and understand nutritional risk among this population.

## 1. Introduction

Population ageing is now a universal phenomenon [1] and women form the majority of older persons, as they currently outlive men by 4.8 years [2]. The population of older adults has been increasing rapidly worldwide as the proportion of people aged 60 and over has increased faster than any other population group due to greater average life expectancy and decreased fertility rates [3,4].

This phenomenon can be seen as a success in the history of public health policies and sociodemographic development, but it is also a societal challenge to adapt policies and programs more focused on identifying strategies to improve the quality of life (QoL), health status, and functional status of individuals in this age group, as well as their social participation and security [5,6]. Older adults present aspects that require a complex assessment with an analysis of risk factors. In addition to several age-related changes in the normal aging process which may affect NS, the literature has been consistent in pointing out multiple factors correlated with malnutrition in older adults such as social, environmental, and health-related factors. In the first instance, age-related changes include a decline in physiological function, sensory impairment such as diminished taste or olfactory dysfunction, delayed gastric emptying, and disturbed motility leading to the functional decline of the gastrointestinal tract. This is combined with a decrease in gastrointestinal hormones, and with adverse changes in anorectic signalling (e.g., leptin, cholecystokinin, neuropeptide Y, peptide YY, orexin A) can lead to a loss of appetite and anorexia of aging, and consequently predispose elders to having insufficient daily nutrient intake, with deleterious outcomes in NS and functionality: weight loss, loss of muscle mass and strength (i.e., sarcopenia), and the frailty syndrome [7,8]. Additionally, NS is affected by multiple interdependent factors, such as lifestyle, loneliness, isolation, marital status, educational level, socioeconomic level, and place of residence [7,9,10,11]. Moreover, sex can impact the risk of malnutrition. Alzahrani et al. recognized that female sex was a significant predictor for malnutrition, since elderly women seem to suffer more often from a lack of appetite and consequent weight loss when compared to their male counterparts [12].

Imbalanced NS presents a huge clinical significance as it adversely affects the health and QoL of older adults, as well as overall healthcare costs. In fact, the consequences of malnutrition have serious implications for clinical and social outcomes, for recovery from disease, and are associated with increased morbidity and mortality both in acute and chronic disease [13].

NS also impairs wound healing and resistance to infection, as it predisposes older adults to wound healing disorders and consequently to chronic wounds which are a great burden as they are associated with decreased QoL, and furthermore result in higher expenditure in the healthcare setting [5,7,11].

The literature highlights that the impact of poor NS is greater in older than in younger adults and occurs to a greater extent, but also that recovery is slower. Malnourished older adults are clearly at higher risk, which in turn greatly compromises their health status, cognitive function, and functionality. In line with this, the assessment of NS should consider several factors, such as the physiological changes of aging and its multifactorial causes, namely social isolation, loneliness, and chronicity of diseases [14].

Despite the growing research interest on NS in older adults, less is known about the elderly living in the community, as they are the group that need health care and social support services the most [11], namely SC. In fact, older adults have poverty rates that are clearly higher than those of the overall population [15], so it is not surprising that elderly people with low incomes are usually the attendees of SCs [16]. SCs are committed to serving poor elderly and minority individuals [16], and are, by definition, places that promote the physical and mental health of community-dwelling elders by providing several services, such as serving lunches, providing a critical community need to seniors who are still active but have limited financial means to make their own lunches, and prefer companionship while eating. Moreover, this setting provides services that satisfy basic needs and psychosocial support, and it promotes socialisation and the occupation of free time, interpersonal relationships, and socio-cultural entertainment in order to avoid isolation. An SC provides a set of services that contribute to the maintenance of the elderly in their social and family environment. In addition, services involve hygiene care, the treatment of clothes, and the service of adapted transport or accompanying services. It is intended, therefore, to have a diversified range of proximity services, allowing the citizen to remain there as long as possible living their usual way of life, thus delaying institutionalization. The motivations to request support from the SC are varied. According to Benet [17], reasons related to health come first when deciding to whether to attend this social facility. Therefore, we conducted this study to evaluate the NS of older adults attending SCs, and to identify its predictors.

## 2. Materials and Methods

### 2.1. Participants and Study Design

Data were collected from September 2015 to February 2016. This is a cross-sectional and observational study conducted in a sample of older adults (aged 65 years or higher) attending SCs in Lisbon. 

**Figure 1:**
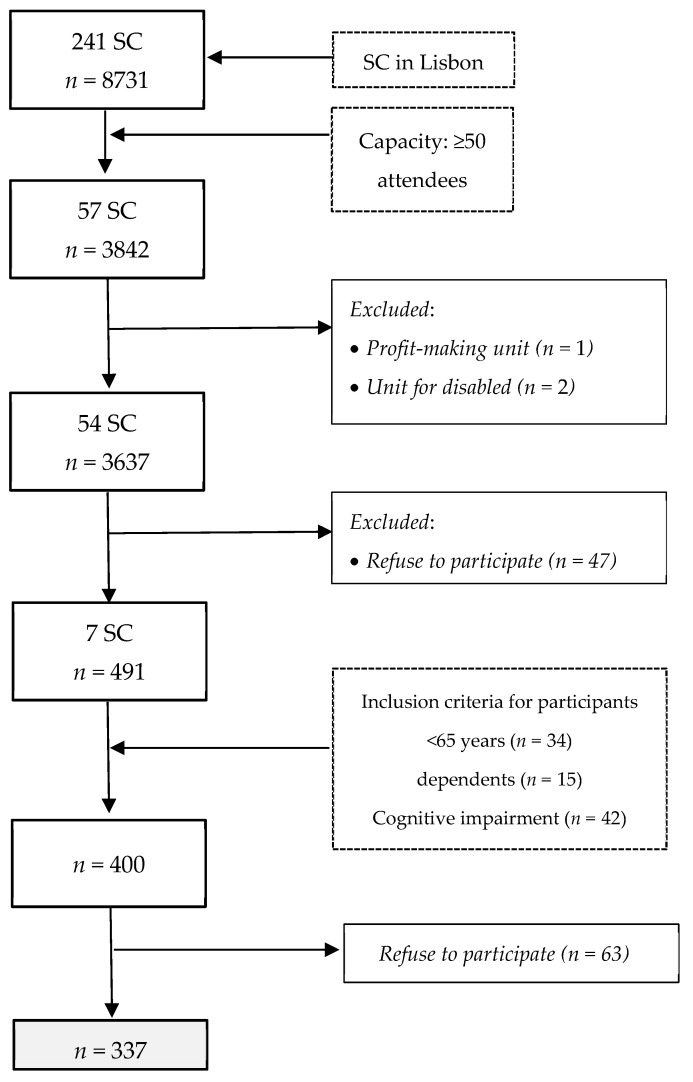
Study design and participants.

In order to select the sample, Carta Social, an online tool describing social equipment in Portugal with financial support from the Ministry of Social Affairs, was used. The inclusion criteria applied for SCs were those belonging to the municipality of Lisbon, and having the capacity for 50 or more attendees. A total of 241 SCs were identified in Lisbon and 54 SCs met the criteria (Figure 1). These SCs were invited to participate by telephone and/or by e-mail, and from those 7 SCs accepted to participate in this study, with a total of 491 attendees. From these, 400 met the criteria and 63 refused to participate. The sample encompassed 337 participants (Figure 1).

The study included participants who met the following criteria: living in the community, being an SC attendee, being 65 years or older, being autonomous in daily living activities, and agreeing to participate in the study through written informed consent. Exclusion criteria were older adults with cognitive impairment that prevented them from understanding and/or answering the questionnaires, or who did not agree to participate in the study. Potential participants were contacted at the SC they were attending, and data were collected through face-to-face interviews in SC facilities by one trained nutritionist.

This investigation was conducted in accordance with the Declaration of Helsinki (World Medical Association, 1983) and approved by the ICBAS-UP Ethics Committee. All ethical issues inherent to the investigative process were safeguarded, and the informed consent of all was obtained. 

Participants were asked sociodemographic and contextual questions, which included sex, age, marital status, education level (number of years spent in school), and number of children. It was also intended to contextualize the institution’s support scheme, duration of the support, and the services they enjoyed, as well as the main reason for attending SCs. 

Health-related data were self-reported: self-reported chronic health problems and multimorbidity, defined as the coexistence of at least two self-reported chronic diseases and alcohol and tobacco consumption; daily medication and the consumption of nutritional/dietary supplements, and restrictions on their use due to economic constraints in the last 12 months at the time of the questionnaire. Finally, participants were asked to classify their health status, with the aim to know their perception about their particular health situation. The scale used (Likert type) presented five possibilities of response: very good, good, fair, poor, and very poor.

### 2.2. Assessment Instruments

To evaluate the social context of the sample, the Gijon Socio-Family Situation Assessment Scale was applied, due to its pertinence and adequacy in order to verify the family situation of the participants and identify the profile of social vulnerability [18].

Mini Nutritional Assessment, commonly referred to as MNA^®^, is a validated tool for assessing the NS of the older adult population [19]. Although there is an abbreviated version, the full form was chosen, as it was considered the most appropriate. It is a questionnaire with a total of 18 items, which include anthropometric assessment, food questionnaire, global assessment, and self-assessment.

All anthropometric measurements were performed following the standard methods (ISAK) [20]. Body weight, height, the calculation and classification of BMI, middle upper arm (MUAC), waist (WC), and calf circumferences (CC) were included. 

Based on the experience of the phenomenon of Food Insecurity (FI), the Food Insecurity Scale (FIES) is already used in several countries to provide reliable information about the ability of individuals and families to access food. The FIES scale contains 8 closed questions, with the possibility of answering yes, no, does not know, and prefers not to answer/refuse, referring to the last 12 months [21].

### 2.3. Cognitive Status

General cognitive function was assessed by Mini-Mental State Examination (MMSE). The most often used screening tool for providing an overall measure of cognitive impairment in clinical, research, and community settings [22], it includes tests of orientation, concentration, attention, verbal memory, naming, and visuospatial skills. The cut-off was set according to the educational level of the participant.

### 2.4. Statistical Analysis

The statistical analysis was performed using SPSS^®^ Statistical Package for Social Sciences (IMB^®^, version 25.0 for Windows).

Basic descriptive data were used to characterize the participants in the study and all variables were assessed for normality. For qualitative variables, absolute frequencies (*n*) and relative frequencies (%) were calculated, while for quantitative variables, the minimum value, maximum value, mean, and standard deviation (SD) were determined as descriptive measures.

Binary logistic regression models (uni- and multivariate) were used to predict malnutrition or risk of malnutrition (assessed using the MNA^®^ and recategorised into a single group), considering participants classified as having a normal NS as the reference group. The independent variables included in the models were sex, age, Gijon’s Socio-Family Situation Assessment score, tobacco or alcohol use, self-perception of health, NCDs, time of SC attendance, reasons for attending the SC, and FI. Self-perception of health was included using good and very good as the reference category, and also combining poor and very poor in one single category. As for FI, food security was defined as the reference category, and moderate and severe FI were also combined into one category. The multivariate regression was adjusted for all variables in the model.

All inferential analyses were performed with 95% confidence.

## 3. Results

### 3.1. Sample Characterization

This study included a total of 337 older adults, with an average age of 78.4 (SD = 7.1 years), between 66 and 99 years, mostly female (62.3%). Females were presented in the majority in all age groups (Table 1). The prevalence of multimorbidity in the total sample was high (96.4%). The average number of self-reported noncommunicable diseases (NCDs) was 4.6 (SD = 1.8). Both sexes presented a high average number of diseases (Table 2). Arterial hypertension (HTN) represented the most predominant pathology (73.9%), and almost the whole sample took daily medication (99.4%) and more than three different medications per day (96.1%).

Most of the participants attended the SC due to a lack of money, loneliness, and conviviality. Almost half of the sample (45.4%) benefited from this support for between 1 and 4 years, and one in ten (10.4%) had been SC attendees for more than 10 years. More than one third of the participants (34.7%) presented high social risk and 70% of the sample presented some level of FI.

Regarding NS, participants at risk of malnutrition represented 40.7% of the sample (Table 3). 

According to Table 4, there are some variables that have a statistically significant relationship (*p* < 0.05) with NS. 

### 3.2. Clinical Factors

When considered separately, the following are predictors of malnutrition or the risk of malnutrition: a worse self-perception of health status (OR = 3.044, CI 95% [1.152–8.048]; *p* = 0.025), having or having had urinary incontinence (OR = 1.646, CI 95% [1.009–2.684]; *p* = 0.046) or depression (OR = 3.594, CI 95% [2.144–6.027], *p* < 0.001). 

According to the adjusted logistic regression model, being older (OR = 1.045, CI 95% [1.003–1.089], having a worse self-perception of health status (OR = 3.395, CI 95% [1.182–9.746], *p* = 0.023), having or having had depression (OR = 5.138, CI 95% [2.869–9.201], *p* < 0.001), and not having or having had respiratory tract problems (OR = 0.477, CI 95% [0.246–0.925], *p* = 0.028) were independent predictors of malnutrition or risk of malnutrition.

### 3.3. Social Factors

In turn, a time of SC attendance equal to or greater than one year but less than five years is associated with lower odds of malnutrition, or risk of malnutrition (OR = 0.506, CI 95% [0.291–0.881], *p* = 0.016).

According to the adjusted model, intermediate attendance times of SC were associated with lower odds of malnutrition or risk (OR = 0.367, CI 95% [0.191–0.705], *p* = 0.003).

## 4. Discussion

It is universal that women have a higher average life expectancy compared to men [23]. Although the mean age between men and women in this study was similar, there was a predominance of female participants. The literature highlights remarkable sex differences in life expectancy and mortality, indicating that human longevity seems to be strongly influenced by sex [23,24]. 

In fact, the aging process is qualitatively different between men and women, since they differ not only biologically (including sex hormones and genetics), but also regarding lifestyles, where nutrition is, along with smoking, physical activity, type of work and education, one of the factors underpinning the sex difference in longevity and aging. Moreover, coping capacity with stressful events (e.g., serving as caregivers or spousal bereavement) also plays a role in this complex lifelong interaction between biological and non-biological factors, which impacts health, the propensity to diseases, and also disabilities later in life [24].

The marital status with the highest representation in the sample was the married state, in which women predominate. Additionally, the majority of widowed participants were women, in line with studies conducted by other authors [25,26], since women live longer as widows than men [26]. It is said that aging in the feminine is more solitary and less conjugal [27]. In terms of education level, we found a considerable prevalence of illiteracy and an average number of years of low education, which would be expected given the low level of education that characterizes this age group in Portugal [25]. 

This sample seems to be a group of older adults with a deterioration of their state of health, as is observed regarding the presence of numerous chronic diseases, reflected in their multimorbidity (96.4%), as well as in polypharmacy, since almost all participants took more than three different medications per day. Other authors also reported that more than 90% of the participants took medications daily [28]. The treatment of NCDs often includes long-term pharmacotherapeutic use [29]. The literature indicates that these elders present a higher risk of nutritional changes, either due to the underlying disease and comorbidities, or due to the therapeutic intervention that the clinical condition requires. In fact, the NS of the elderly interferes with the pharmacokinetics and pharmacodynamics, potentiating side effects. Additionally, the taking of drugs can also conditionate NS [30,31]. Prescribing drugs from different pharmaceutical groups also increases the risk of drug interactions that may result in negative outcomes.

More than half of the sample had normal NS, while 40.7% were at risk of malnutrition and approximately 5% were malnourished. The latter value is in accordance with the literature, which estimates that, in the community, malnutrition values in the elderly are between 0.7% and 5.8% [29].

Older adults present different aspects, which require a broader assessment with the analysis of risk factors, such as the physiological changes of aging and the multifactorial causes of poor NS, which include social isolation, loneliness, chronicity of diseases, changes in psychological disorders, depression, and disabilities [14,32]. Thus, these results reinforce the importance of nutritional assessment in the comprehensive assessment of the elderly (also known as Comprehensive Geriatric Assessment—CGA) [33], and intensify the need for nutritionists in the multidisciplinary teams that accompany the elderly, mainly for a rigorous and precise assessment of NS and an individualized nutritional prescription.

The predictors of NS are varied, and include clinical status and socioeconomic parameters [11,34]. It is well stablished that older adults are more vulnerable to malnutrition than the young, due to the presence of several chronic diseases [35,36], for which age is the greatest risk factor [37]. In fact, malnutrition is widespread among older adults. Due the rapid increase in this age group and their high risk of dependence, identifying factors that allow them to maintain a healthier and more independent life is of prime interest [37]. Age is recognized as one of the main risk factors for NS in the elderly [36,38], and, in this research, age constitutes an independent predictor of malnutrition or risk. The odds of having malnutrition or risk increased by 1.045 per year in the sample. 

In addition to the numerous age-related changes during the normal aging process that can affect NS [39], the susceptibility of the elderly is related to their vulnerability and various disabilities [40], presence of chronic diseases [41], low income, and need for assistance with DLAs [36]. The set of these factors is often intertwined with the presence of FI, which negatively affects health, QoL and NS [35], as well as mental health [42,43], as it leads to depression and contributes to a more rapid decline in cognitive function [27,44,45]. Thus, older adults are at increased risk of malnutrition due to a variety of psychological, physiological and social factors [46]. The existence of at least one non-communicable chronic disease (NCD) increases from the age of 65 [47]. In fact, the literature highlights the high prevalence of multiple chronic diseases among the elderly [15,48,49]. With regard to self-reported chronic diseases and lifestyles, it was found that the most representative diseases in the sample were arterial hypertension (AHT), degenerative joint diseases, and hypercholesterolemia. These data are corroborated in national studies: since 1998, AHT and back pain have been presented as the most frequent chronic diseases among the elderly population surveyed [46]. Additionally, the National Health Survey 2005/2006 [48] highlighted that the elderly were the population group with the highest prevalence of chronic diseases, in particular HTN, and with situations of temporary or permanent disability, such as rheumatic disease, chronic pain, and diabetes, which registered a higher prevalence among the elderly, as observed among the participants of this study. In 2014, the most prevalent chronic diseases among the Portuguese elderly remained unchanged. Likewise, recent data from the national study Epidoc, with elderly people, corroborate the results of the present investigation [48].

Self-reported health status is a predictor of morbidity and mortality in older adults [50]. The association between poorer self-perceived health status and higher risk of malnutrition has been reported [51]. In the present investigation, a better self-perceived state of health decreased nutritional risk and malnutrition. The same was corroborated by Simsek et al. [11].

Regarding health variables, not having respiratory tract diseases decreases the probability of these older adults having a risk of malnutrition, in line with other studies; respiratory diseases, namely pulmonary emphysema, are predictors for malnutrition in the elderly population [52]. Urinary incontinence proved to be a predictor of malnutrition or risk when considered alone, but loses statistical significance in the adjusted models, contrary to other studies [53].

Nutrition may play a protective role against several health conditions, including cognitive decline [54]. In fact, depression, very prevalent in elders [55], has been reported as a major determinant for malnutrition in the elderly [56]. In agreement with the results of the present investigation, the elderly who reported depression were more likely to have malnutrition or to be at risk. Thus, depression is strongly associated with poor NS [57]. Additionally, depression is a predictor for FI [58], which in turn is an important risk factor in the elderly [59]. The loss of an active social life, and the deprivation of affection, so common in situations of loneliness, can induce a state of depression that can arise clinically as anorexia [34]. The elderly can perceive reality with a sense of futility and live with a passive attitude and disinterest. It should be noted that the first manifestation of depression is the lack of interest in food [50]. In the sample, depression is related to NS, being an independent predictor for malnutrition and the risk of malnutrition.

Participants who had been attending an SC for less than 5 years were 3.7 times less likely to have malnutrition or nutritional risk, being an independent factor. We can hypothesize that the SC contributes not only to guaranteeing the basic right to food—since the elderly may not be able to prepare balanced meals due to disabilities or lack of money—but also contributes to promoting social relationships with other attendees, thus reducing social isolation. Loneliness, social isolation, and other social aspects are important among elders.

Its impact on health and particularly on NS and the risk of this population is multifactorial [54,60]. Ramic and colleagues also explored the effect of loneliness on malnutrition in the elderly, concluding that loneliness was a strong predictor for the risk of malnutrition [61]. This condition can be explained by the negative effects on appetite and decreased nutrient intake due to mood and functional and cognitive decline [54], promoting an inability to buy, prepare, and make food, or even leading to the person forgetting to eat [34,54]. This disability creates a vicious cycle where malnutrition and functional and mental deterioration sustain and reinforce each other [34,50]. Thus, these elderly people should not eat their meals alone [54]. Active social participation is an important indicator of QoL in adulthood and overlaps with age-related losses and physical, mental, and social changes, serving as a protective factor against depression [62]. Having little social and material support can discourage the individual from spending time on buying healthier and also less expensive food options, which require preparation and some knowledge; so, social support positively influences healthy behaviours [63], as well as provides a “safety net” to which the elderly can turn whenever necessary. In the same vein, Sahyoun and Zhang (2005) reported that social interaction and food are positively associated with each other [62].

The reason “loneliness” for attending an SC was not shown to be a predictor in this sample. However, some literature on the association between social isolation and subjective loneliness and malnutrition in the elderly has shown a relationship between them, suggesting that social isolation and subjective loneliness are two independent risk factors for malnutrition in the elderly population [60]. Nevertheless, it seems pertinent to conclude that the SC can be a protective entity of the elderly who attend this social response. Another aspect that deserves discussion is the fact that the deterioration of the NS is closely related to lack of food intake, weight loss, disease, functional status, and stress [64], which is pointed out in the literature as a biopsychosocial factor for NS [52,65,66]. Both psychological stress and acute illness are threats to homeostasis [67], particularly acute disease is very catabolic and may promote unintentional weight loss and energy-protein malnutrition. Contrary to the literature revisited, in the present study, the insecure elderly were not more likely to be malnourished. These results do not reinforce the results found in other investigations [35,68]. Still, FI and malnutrition in the elderly should be analysed together, as economic deprivation is related to FI and also to malnutrition. Social factors such as poverty, loneliness, and low education level can also affect food availability and consequently the NS [21,60]. This binomial is complex, bidirectional, and self-perpetuating, as FI leads to a worse state of health and worse NS, which in turn leads to an increase in the severity of the FI, and thus the cycle continues [68,69,70,71]. The problem of FI is distinct in this age group and is assumed to be a risk factor for malnutrition in the elderly [36]. The fact that the whole sample includes users of SCs who eat in these institutions may explain this result, meaning that properly oriented SCs may have a crucial and protective role in the NS of these elders. Limited access to food contributes to a further deterioration in health status [46], and puts the elderly at nutritional risk [42].

## 5. Limitations and Strengths

Some limitations should be considered. The cross-sectional design of the study does not allow causality inference. The clinical data (namely NCDs) were self-reported and may therefore be biased, as there might be an underestimation of some health conditions due to a lack of memory, highly prevalent in older adults. Additionally, the sample encompassed older adults living in the community in a specific Portuguese region. Thereby, more studies with similar methos should be performed among other populations to verify the possibility of their generalization. Another limitation concerns the exclusion criteria of participants with cognitive impairment that may have biased some results, since many studies reported a relationship between cognitive impairment and a higher prevalence of malnutrition or risk of malnutrition. 

However, the present study was strengthened using appropriate methodology and validated tools. There was only one interviewer for the entire sample, which prevented the existence of interindividual variations. 

To search for malnutrition-related features (as assessed by the MNA items) and identify predictors based on sociodemographic, clinical data, and anthropometric measurements can provide crucial information for the development of direct and precise interventions, leading to overcoming nutritional risk, and this may contribute to early assessment and intervention in this specific population.

## 6. Conclusions

In conclusion, this study indicates that older age, worse self-perception of health status, depression, and the absence of respiratory tract problems were independent factors of malnutrition or risk. An SC attendance time of up to 5 years was shown to be an independent predictor of the lower odds of having malnutrition or nutritional risk. Therefore, keeping the elderly integrated into society, participatory and active, satisfied with their health status and perceiving it as positive can be protective behaviours for maintaining NS, in which the SC seems to be a mediator. Due to the above, the results support the fact that NS in older adults has a multifactorial aetiology, with a strongly social component, and is related to health circumstances. From this point of view, the evaluation of the elderly should integrate a comprehensive approach and consider all the dimensions of the concept of health. Further studies are needed to explore and understand nutritional risk among this population. Early screening to identify nutritional risk factors may allow for timely multimodal intervention and thus better clinical outcomes.

## Figures and Tables

**Table 1:** Distribution of age groups by sex, marital status, and education.

	Total		Female		Male	
Age (mean ± SD)	78.4 (±7.1)		78.6 (±7.0)		77.9 (±7.1)	
Max–Min	66–99		97–66		99–66	
	*n*	%	*n*	%	*n*	%
Age groups						
65 to 74 years (Young-old)	111	32.9	74	66.7	37	33.3
75 to 84 years (Middle-old)	156	46.3	95	60.9	61	39.1
≥85 years (Old-old)	70	20.8	41	58.6	29	41.4
Marital status						
Single	6	1.8	4	1,9	2	1,6
Married	157	46.6	88	41.9	69	54.3
Divorced	28	8.3	19	9.0	9	7.1
De facto union	5	1.5	3	1.4	2	1.6
Widow/widower	141	41.8	96	45.7	45	35.4
Education Level						
0 years	35	10.4	23	11	12	9.4
1 to 5 years	270	80.1	166	81	100	78.6
6 to 10 years	26	7.8	13	6.2	13	10.3
More than 10 years	6	1.8	4	1.9	2	1.6
Total	337	100	210	62.3	127	37.7

**Table 2:** Multimorbidity and NCDs.

	Female		Male		Total	
	*n*	%	*n*	%	*n*	%
Without multimorbidity	5	2.4	7	5.5	12	3.6
With multimorbidity	205	97.6	120	94.5	325	96.4
Daily medication	210	100	125	98.4	335	99.4
More than 3 different medications per day	204		120		324	96,1
Total	210	100	127	100	337	100
Number of NCDs (mean ± SD)	4.7 ± 1.8		4.4 ± 1.8		4.6 ± 1.8	
NCDs	*n*	%	*n*	%	*n*	%
Allergies	32	15.2	20	15.7	52	15.4
Does not know	1	0.5	0	0.0		
Autoimmune Diseases	19	9.0	5	3.9	24	7.1
Stroke	40	19.0	25	19.7	65	19.3
Degenerative Joint Diseases	159	75.7	75	59.1	234	69.4
Depression	50	23.8	38	29.9	88	26.1
Diabetes	64	30.5	27	21.3	91	27
Coronary heart disease/angina pectoris	22	10.5	22	17.3	44	13.1
Acute myocardial infarction	18	8.6	26	20.5	44	13.1
Liver diseases	0	0.0	3	2.4	3	0.9
Hypercholesterolemia	133	63.3	80	63.0	213	63.2
Hypertension	157	74.8	92	72.4	249	73.9
Urinary incontinence	63	30.0	25	19.7	88	26.1
Chronic renal failure	17	8.1	6	4.7	23	6.8
Oncological diseases	38	18.1	14	11.0	52	15.4
Respiratory diseases	32	15.2	23	18.1	55	16.3
Diseases of the Gastrointestinal Tract	79	37.6	43	33.9	122	36.2
Other(s)	65	31.0	28	22.0	93	27.6

**Table 3:** Nutritional Status (MNA), by sex.

Nutritional Status Assessment	Female		Male		Total	
	*n*	%	*n*	%	*n*	%
Malnourish (<17 points)	14	4.2	2	0.6	16	4.7
At risk (17–23.5 points)	87	25.8	50	14.8	137	40.7
Normal (24–30 points)	109	32.3	75	22.3	184	54.6
Total	210	62.3	127	37.7	337	100

**Table 4:** Predictors of malnutrition and nutritional risk.

			Models					
		*n*	Non Adjusted			Adjusted		
			OR	IC95%	*p*	OR	IC95%	*p*
Sex	Fem. (ref. male)	210 (ref. 127)	1.336	0.856; 2.086	0.202			
Age	(years)	337	1.016	0.986; 1.048	0.306	1.045	1.003; 1.089	0.037
Gijon Socio-Family Situation Assessment Scale	(total score)	337	0.952	0.857; 1.059	0.365			
Tobacco	Yes (ref. no)	23 (ref. 314)	0.759	0.319; 1.805	0.533			
Alcohol	Yes (ref. no)	62 (ref. 314)	1.070	0.616; 1.860	0.810			
Self-perception of health	good/very good (ref.)	23			0.013			0.016
	Fair	119	1.785	0.656; 4.859	0.256	1.948	0.660; 5.749	0.227
	poor/very poor	195	3.044	1.152; 8.048	0.025	3.395	1.182; 9.746	0.023
Respiratory tract problem	Yes (ref.: no)	55 (ref.: 282)	0.583	0.319; 1.065	0.079	0.477	0.246; 0.925	0.028
Hypercholesterolemia	Yes (ref.: no)	213 (ref.: 124)	0.785	0.504; 1.225	0.286			
Myocardial infarction	Yes (ref.: no)	44 (ref.: 293)	1.888	0.992; 3.594	0.053			
Coronary heart disease/angina pectoris	Yes (ref.: no)	44 (ref.: 293)	1.373	0.728; 2.591	0.327			
Arterial hypertension	Yes (ref.: no)	249 (ref.: 88)	0.732	0.450; 1.192	0.209			
Stroke	Yes (ref.: no)	65 (ref.: 272)	1.645	0.954; 2.836	0.073			
Degenerative joint diseases	Yes (ref.: no)	234 (ref.: 103)	0.788	0.495; 1.254	0.315			
Autoimmune disease	Yes (ref.: no)	24 (ref.: 313)	0.849	0.366; 1.970	0.703			
Oncological disease	Yes (ref.: no)	52 (ref.: 285)	1.637	0.903; 2.969	0.105			
Diabetes mellitus	Yes (ref.: no)	91 (ref.: 246)	1.042	0.643; 1.689	0.866			
Allergies	Yes (ref.: no)	52 (ref.: 285)	0.714	0.390; 1.308	0.276			
Renal failure	Yes (ref.: no)	23 (ref.: 314)	1.110	0.476; 2.592	0.809			
Urinary incontinence	Yes (ref.: no)	88 (ref.: 249)	1.646	1.009; 2.684	0.046			
Gastrointestinal diseases	Yes (ref.: no)	122 (ref.: 215)	0.980	0.627; 1.531	0.929			
Depression	Yes (ref.: no)	88 (ref.: 249)	3.594	2.144; 6.027	<0.001	5.138	2.869; 9.201	<0.001
Suspended medication	Yes (ref.: no)	181 (ref.: 156)	1.203	0.782; 1.850	0.401			
Time of SC attendance	<1 year (ref.)	78			0.007			0.002
	[1; 5[ years	153	0.506	0.291; 0.881	0.016	0.367	0.191; 0.705	0.003
	≥5 years	106	1.050	0.584; 1.886	0.871	0.796	0.372; 1.704	0.557
Reason: money	Yes (ref.: no)	245 (ref.: 92)	1.047	0.647; 1.696	0.850			
Reason: loneliness	Yes (ref.: no)	71 (ref.: 266)	1.625	0.960; 2.752	0.071			
Reason: cooking	Yes (ref.: no)	18 (ref.: 319)	1.538	0.592; 4.000	0.377			
Reason: conviviality	Yes (ref.: no)	86 (ref.: 251)	0.997	0.610; 1.631	0.991			
FI	Food Security (ref.)	101			0.484			
	Mild FI	117	0.897	0.524; 1.535	0.691			
	moderate/severe FI	119	1.224	0.719; 2.083	0.457			

Adjusted model: step-by-step advance method (likelihood ratio); Nagelkerke’s R^2^ = 0.210; *p* < 0.001; *n*: normal = 184; at risk + malnutrition = 153.

## Data Availability

All raw data will be available upon reasonable request to the corresponding authors.
